# Exchange of cellular components between platelets and tumor cells: impact on tumor cells behavior

**DOI:** 10.7150/thno.64252

**Published:** 2022-02-07

**Authors:** Alba Rodriguez-Martinez, Iris Simon-Saez, Sonia Perales, Carmen Garrido-Navas, Alessandro Russo, Diego de Miguel-Perez, Ignacio Puche-Sanz, Clara Alaminos, Jorge Ceron, Jose A. Lorente, Maria Pilar Molina, Coral Gonzalez, Massimo Cristofanilli, Alba Ortigosa-Palomo, Pedro J. Real, Christian Rolfo, María J. Serrano

**Affiliations:** 1 GENYO, Centre for Genomics and Oncological Research: Pfizer/University of Granada/Andalusian Regional Government, Granada, Spain.; 2 Laboratory of Genetic Identification, Department of Legal Medicine, University of Granada, Granada, Spain.; 3 Department of Biochemistry and Molecular Biology I, Faculty of Science, University of Granada, Granada, Spain.; 4 Center for Thoracic Oncology. The Tisch Cancer Institute. Icahn School of Medicine at Mount Sinai, New York, NY, USA.; 5 Urology Department, University Hospital Virgen de las Nieves, Granada, Spain.; 6 Urology Division, University Hospital of Jaén, Jaén, Spain.; 7 Robert H Lurie Comprehensive Cancer Center, Northwestern University, Chicago, IL, USA.; 8 Instituto Biosanitario Granada (iBS-Granada), Granada, Spain.; 9 Integral Oncology Division, Virgen de las Nieves University Hospital, Granada, Spain.; 10 Department of Pathological Anatomy, Faculty of Medicine, University of Granada, Granada, Spain.

**Keywords:** Platelets, circulating tumor cells (CTCs), platelet-educated tumors (PETs), epithelial-to-mesenchymal transition (EMT), CD61

## Abstract

**Background:** Platelets are active players in tumorigenesis, although the exact interactive mechanisms and their direct impact on tumor cells remain largely unknown.

**Methods:** Bidirectional transference of lipids, proteins and RNA between platelets and tumor cells and its impact on tumor cell behavior and tumor process are analyzed in this work. Phenotypic, genetic and functional modifications induced by platelets were analyzed both in tumor cell lines and in circulating tumor cells (CTCs).

**Results:** Data from these assays showed that platelets transferred structural components to tumor cells with higher efficiency than tumor cells to platelets (p = 0.001). This biological interplay occurred by direct contact, internalization or via extracellular vesicles. As a result, tumor cells acquired platelet markers (CD61 and CD42), showed decreased EpCAM, expressed epithelial-to-mesenchymal transition markers, and increased proliferation rates. Moreover, we were able to detect CD61 in CTCs from early and advanced prostate cancer.

**Conclusions:** Our results demonstrated, for the first time, that platelets educate tumor cells by highly efficient transference of lipids, proteins and RNA through different mechanisms. These results suggest that tumor cells and CTCs might acquire highly dynamic and aggressive phenotypes due to platelets interaction including EMT, stem-like phenotype and high proliferative rates.

## Introduction

The tumor dissemination process involves release of tumor cells from the primary site to the bloodstream or the lymphatic system, known as circulating tumor cells (CTCs) 1. Albeit, it is widely accepted that the presence of CTCs in peripheral blood of cancer patients is strongly associated with poor survival outcomes 2, little is known about their biological complexity. Each day millions of tumor cells are released from the tumor site into the blood, but only few of them survive 3. CTCs are subjected to a combination of physical stress (shear forces) 4, anoikis (a form of cell death that occurs in anchorage-dependent cells when they detach from the surrounding extracellular matrix) 5, and are exposed to the immune system activity 6. CTC survival may depend on their physical and molecular adapting ability which involves, presence of different CTCs subpopulations with markedly distinct characteristics 7.

The immune system has a dual role in cancer progression with both repressive and promoting actions. The establishment of CTCs clusters or microemboli, composed of CTCs, leukocytes, cancer-associated fibroblasts, endothelial cells and platelets, facilitates tumor immunoescape 8,9 enabling metastasis 10. Besides their physiological function in homeostasis, platelets have been shown to serve as active players during carcinogenesis, through mechanisms that affect both CTC migration and survival in circulation 9,11,12. A complex crosstalk between cancer cells and platelets exists and the exact underlying mechanisms are relatively poor understood. Platelets are able to sequester tumor RNAs, turning into tumor-educated platelets (TEPs) 13. Since TEPs discovery, numerous studies have analyzed the modalities for which tumor cells can modify platelets converting them into a potential predictive and prognostic biomarker in cancer through the evaluation of liquid biopsies 14. However, few works have focused on investigating the mechanisms by which tumor cells are directly modified by platelets 15 and how this interaction affects their structural composition and therefore their phenotype and consequently their functionality 16. The most widely studied consequence of the interaction between tumor cells and the immune system is the acquisition of mesenchymal phenotypes 17 through induction of the epithelial mesenchymal transition process (EMT) 18,19.

Herein, we explored the tangled bidirectional interactions between platelets and tumor cells, revealing that platelets can actively modify tumor cells phenotype, their genetic content and their functional abilities.

## Methods

### Sample collection

Peripheral blood samples from both healthy donors (with no history of malignant disease) and prostate cancer patients were obtained from Virgen de las Nieves University Hospital (Granada) after approval by the ethical Committee of this Hospital, in accordance with the Declaration of Helsinki. Written informed consent was signed from every cancer patient and healthy volunteer prior sample collection.

Samples were processed in the Liquid Biopsies & Cancer Interception laboratory (LiqBiopCI) at GENYO Centre (Granada). All prostate cancer patients were diagnosed and followed-up in the Urology Department and in the Oncology Department of the University Hospital Virgen de las Nieves (Granada).

### Circulating Tumor Cells (CTCs) isolation

Peripheral blood samples (10 mL) from prostate cancer patients diagnosed of localized disease or advanced disease (metastatic castration resistant prostate cancer) were collected in EDTA tubes (Vacutainer), stored at room temperature and processed into 4 h after collection. CTCs were isolated according to the previously established protocol by our group 20,21. Briefly, blood samples were subjected to density gradient centrifugation and immunomagnetic selection of epithelial cells using the Carcinoma Cell Enrichment and Detection Kit (Miltenyi Biotec) based on pan-cytokeratin (CK3-11D5) microbeads. Each sample was spun down onto two slides in a cytocentrifuge (Hettich) and stained for confocal microscopy visualization.

### Detection and characterization of CTCs from Prostate Cancer Patients

Slides from prostate cancer patients CTCs isolation and fixed cells were stained with mouse anti-human Cytokeratin-FITC (Ref. 130-119-141, Miltenyi), mouse anti-human CD61-Alexa Fluor 647 (Ref. 336408, Biolegend), and Hoechst 33342 (Thermo Fisher). Both sample types were mounted with SlowFade™ Antifade Kit (Invitrogen), for confocal microscopy analysis as previously described. Negative and single stained controls were performed to ensure no fluorescence bleed-through between channels. CTCs were described as CK^+^ nucleus^+^ cells and CD61 expression in CTCs was classified into presence or absence.

### Platelet isolation

Platelets were isolated from whole blood collected in EDTA tubes (Vacutainer) by a series of centrifugations at room temperature in a swing-bucket rotor centrifuge. First, leukocyte-rich platelet-rich plasma (L-PRP) was obtained by centrifugation at 120 × g for 10 min without break. Then, remaining white blood cells and erythrocytes were removed by centrifugation at 105 × g for 15 min to obtain pure platelet-rich plasma (P-PRP). Platelets were isolated from P-PRP by centrifugation at 1000 × g for 12 min. Isolated platelets were resuspended in RPMI 1640 at physiological concentration. Experiments were performed immediately after platelet isolation.

### Platelet activation induction

Platelet activation was induced by incubation with 15 µg/mL of Adenosine 5'-diphosphate sodium salt (ADP) (Sigma-Aldrich) and 1 U/mL of thrombin from bovine plasma (Sigma-Aldrich) for 30 min. Activated platelets were washed with PBS-EDTA 2 mM and transferred to the cell culture.

### Cell tracking

Interactions between platelets and cells were evaluated by labeling either cells or isolated platelets using membrane cell tracker Vybrant™ DiO Cell-Labeling Solution (emission max. 501 nm, green) (Ref. V22886, Invitrogen) or Vybrant™ DiD Cell-Labeling Solution (emission max. 665 nm, red) (Ref. V22887, Invitrogen) at 5 μM final concentration for 20 min at 37 °C. RNA transference was labeled by incubation with Syto RNASelect™ Green Fluorescent Cell Stain Solution (emission max. 530 nm) (Ref. S32703, Life Technologies) at 500 nM (cells) or 10 μM (platelets) for 20 min at 37 °C. Cells and platelets were washed twice with culture media after staining. Alternative staining of cells or platelets before co-culture allowed the measurement of cell tracker transference from platelet to cells and from cell to platelets.

### Cell culture

Human cancer cell lines were used according to different tumor types: LNCAP, PC3 and 22RV1 (from prostate cancer); H1975, H1299 and, A549 (from lung cancer); SW480 and SW620 (from colon cancer), and MCF7, MDA-MB-231, and HCC70 (from breast cancer). LNCAP, PC3, 22Rv1, H1975, H1299, SW480, and SW620 were cultivated in RPMI 1640 (BioWest) while A549, MCF7, MDA-MB-231, and HCC70 were maintained in Dulbecco's minimal essential medium (DMEM) (BioWest). Both media were supplemented with 10% of Fetal Bovine Serum (FBS) (BioWest), 100 U/mL penicillin and 100 mg/mL streptomycin. Cells were maintained at 37 °C in a humidified incubator infused with 5% CO_2_.

Cell lines used in this study were obtained from the American Type Culture Collection (ATCC) and Centre for Scientific Instrumentation (CIC) of the University of Granada; PC3 was kindly donated by Dr. Ignacio Gil Bazo (CIMA, Pamplona). Cell lines were routinely tested for mycoplasma contamination using the Venor®GeM qEP (Minerva Biolabs) and authenticated using AmpFLSTR® Identifiler® Plus (Applied Biosystem). Cells used in the study were mycoplasma free and STR validated.

Most relevant information about Material and Methods is described below and graphically represented in Figure S1. Additionally, functional experiments are described in Supplementary Information.

### Co-culture

For all experiments including tumor cell and platelets co-culture, tumor cells were seeded the day before platelet isolation in order to reach 60-70% of confluence at the time of co-culture. In experiments avoiding direct contact between cells and platelets, 0.4 μm membrane Transwell® inserts (Millipore) were placed on well plates and platelets were added onto them.

### Flow cytometry

Flow cytometry experiments were performed using 24-well plates and 500 μL of platelets suspension. Experiments were run in triplicates and collected at different time points. After co-culture, platelets were harvested from cell media after centrifugation at 105 × g for 15 min to eliminate cell fragments. Cells were washed twice with PBS 1 X to remove any remaining platelets and cell colonies were dissociated with Tryple Express 1 X (Life Technologies). Cells and platelets suspensions were fixed with 3.7% paraformaldehyde (PFA) (Electron Microscopy Sciences) for 20 min at room temperature.

For the analysis of cell activation, platelets were incubated with mouse anti-human PAC-1 (Ref. 340535, BD Pharmingen), and subsequently with goat anti-mouse Alexa Fluor 647 (Ref. A32728, Invitrogen) secondary antibody. After that, platelets were stained with mouse anti-human CD41a-PE (Ref. 555467, BD Pharmingen) for 30 min at room temperature.

In cell tracker transference related experiments, fixed cells and platelets were incubated for 30 min with mouse anti-human CD42b-APC (Ref. 551061, BD Pharmingen), mouse anti-human CD61-Alexa Fluor 647 (Ref. 336408, Biolegend), and rabbit anti-human EpCAM (Ref. ab225894, Abcam). Subsequently, cells and platelets were incubated with goat anti-rabbit Dylight 405 (Ref. 35551, Invitrogen) secondary antibody for 30 min at room temperature.

Between primary and secondary antibody incubations, cells and platelets were washed with FACS Buffer (PBS 1 X, 5% FBS, EDTA 2Mm) and PBS-EDTA 2 mM, respectively.

Both cells and platelets were analyzed in the BD FACSVerse™ flow cytometer equipped with three lasers: violet (405 nm), blue (488 nm) and red (633 nm) (BD Bioscience) using BD FACSuite™ software (BD Bioscience) for acquisition or by FACS ARIA III™ flow cytometer equipped with four lasers: violet (405 nm), blue (488 nm), yellow/green (531 nm) and red (633 nm) (BD Bioscience) using BD FACSDiva™ software (BD Bioscience) for acquisition and FlowJo™ for analysis (FlowJo, LLC-BD Bioscience). Flow cytometry gating strategy is described in Figure S2.

### Confocal Microscopy

To study transference mechanisms, cells were seeded onto Poly-L-Lysine (Sigma Aldrich) pre-treated cover slides. Vybrant™ DiO Cell-Labeling Solution (emission max. 501 nm, green) (Invitrogen) and Vybrant™ DiD Cell-Labeling Solution (emission max. 665 nm, red) (Invitrogen) were used to label platelets and cells respectively.

Transference of RNA was visualized labeling platelets with 10 μM Syto RNASelect™ Green Fluorescent Cell Stain Solution and 5 μM Vybrant™ DiD Cell-Labeling Solution (emission max. 665 nm, red) (Invitrogen) to stain platelet membrane.

After co-culture, platelets were aspirated, and cells were washed once with DPBS Ca^+^ Mg^+^ (Gibco), fixed with 3.7% of PFA and stained with Hoechst 33342 (Thermo Fisher). Cover slides were mounted with SlowFade™ Antifade Kit (Invitrogen).

Time-Lapse Assay were performed in Glass Bottom 35 mm µ-dish (Ibidi). Tumor cells were labeled with DiD cell tracker (red) and platelets with DiO cell tracker (green), as previously described. Platelets were added at 0 time point and set in the incubation chamber of the confocal microscope at 37 °C and 5% CO_2_ for time lapse monitoring. Five positions were analyzed and a total of 24 images were acquired with 10-min/image-time interval for a total duration of 240 min.

Confocal images were obtained using a LSM 710 confocal laser scanning microscope (Carl Zeiss, Jena, Germany) equipped with an incubation chamber (Pecon, Germany). Images were acquired with a Zeiss Plan-Apochromat 63×/1.40 NA DIC M27 oil-immersion objective and ZEN 2010 software (Carl Zeiss, Jena, Germany). Cells were excited with a 405 nm diode laser line, a 488 nm argon laser line, a 543 nm HeNe laser line and a 594 HeNe laser line.

### Electron Microscopy

Monolayer cell cultures of LNCAP cells and platelets were conducted in 8 wells Permanox Lab-Tek^®^ Chamber Slides (NUNC) and fixed at 4 °C in 1.5% glutaraldehyde, 1% formaldehyde, 0.05 M cocodilate buffer. Fixed cells were post-fixed in 1% osmium tetroxide for 1 hour at 4 °C, washed in distilled water, and treated with 0.15% tannic acid and 2% uranyl acetate. Then, dehydration through graded alcohols and propylene oxide, and then embedding in EMbed (Electron Microscopy Sciences) was done. Ultrathin sections (50-70 nm) were stained with 1% uranyl acetate and lead citrate 22. Samples were prepared and examined in a Transmission Electron Microscope using the Libra 120 (Zeiss) ITEM Imaging Platform Software (Olympus) at the Centre for Scientific Instrumentation (CIC) of the University of Granada.

### Image Stream

After co-culture, platelets and LNCAP cells were collected as previously described for antibody staining, after which cells and platelets were fixed using the FIX & PERM^®^ Cell Permeabilization kit (Invitrogen) following manufacturer instructions. Fixed cells and platelets were incubated for 30 min with mouse anti-human CD42b-APC (Ref. 551061, BD Pharmingen). Platelets were also incubated with rabbit anti-human EpCAM (Ref. ab225894, Abcam) for 30 min and subsequently incubated with goat anti-rabbit Dylight 405 (Ref. 35551, Invitrogen) secondary antibody for 30 min at room temperature. Cell DNA was labeled by cell resuspension in 1 X of Hoechst 33342 (Thermo Fisher) and incubation for 5 min. Finally, platelets and cells were resuspended in FACS Buffer for cells and PBS-EDTA 2 mM for platelets. Both cells and platelets data were acquired in an ImageStream® Mark II Imaging Flow Cytometer with four lasers; violet (405 nm), blue (488 nm), yellow (561 nm) and red (642 nm) (Amnis) and analyzed with the software IDEAS.

### Gene Expression

Total RNA from cancer cells cultured alone or in co-culture with non-activated platelets was extracted with TRIzol^TM^ Reagent (Invitrogen), according to manufacturer's instructions. RNA concentration and purity were determined using NanoDrop 2000c Spectrophotometer (ThermoFisher Scientific) and 1 µg of total RNA was converted to complementary DNA (cDNA) using the Transcriptor First Strand cDNA Synthesis Kit (Roche) for subsequent RNA expression.

qRT-PCR primers previously described elsewhere were used (Sigma-Aldrich), details in Table S1. Gene expression was measured using iTaq™ Universal SYBR® Green Supermix (Biorad) on a 7900 HT Real-Time PCR system (Life Technologies). Each test was run three times including non-template controls (NTC). GAPDH was selected as endogenous control. Expression levels are shown as 2^-ΔΔCt^ paired for control and co-cultured cells for normalization at selected time points (1 h, 24 h and 48 h).

### Statistics

Statistical analyses and graphs were performed using IBM SPSS Statistics (version 22.0 for Windows, IBM Corp.) and GraphPad Prism (version 7.04 for Windows, GraphPad software). All expression experiments and functional experiments were performed in triplicates. One-way ANOVA and Two-way ANOVA (Multiple comparisons test) were used. Two-tailed unpaired *t*-test, was performed. CTCs were assessed as a continuous (number of CTCs) and CD61 expression was defined as a dichotomous variable (positive or negative). Dynamics of tumor cell growth after platelets addition were studied using non-linear regression (second order polynomial, quadratic) best-fit modeling, moreover tumor cell growth with or without platelets were compared at 24 h and 48 h. *P* values less than 0.05 were considered statistically significant.

## Results

### Platelets co-culturing with tumor cells leads to platelets activation

First, we analyzed the effect of platelets isolation procedure (P_0_), 24 h culture (P), cell tracker labeling (P_CT_) and ADP+Thrombin treatment (P_ADP+T_) on platelet activation. PAC-1 staining demonstrated that platelets were not activated during isolation (P_0_) neither after 24 h in culture (P). PAC-1 levels were significantly increased in platelets activated with ADP+T (P_ADP+T_) (*p* < 0.01) and labelled with cell tracker (P_CT_) (*p* < 0.05) (Figure S3A).

Second, platelets activation in different co-culture conditions with cells (C) was analyzed. We observed a significant increase in PAC-1^+^ platelets in all conditions: unlabeled co-culture (P+C) (*p* < 0.001), labeled co-culture independently on the labeled cell type (P+C_CT_ and P_CT_+C) (*p* < 0.01 and *p* < 0.001, respectively), and when ADP+Thrombin-treated platelets were used (*p* < 0.05) (Figure S3A).

### Communication between platelets and tumor cells through lipid membrane components

**Figure 1:**
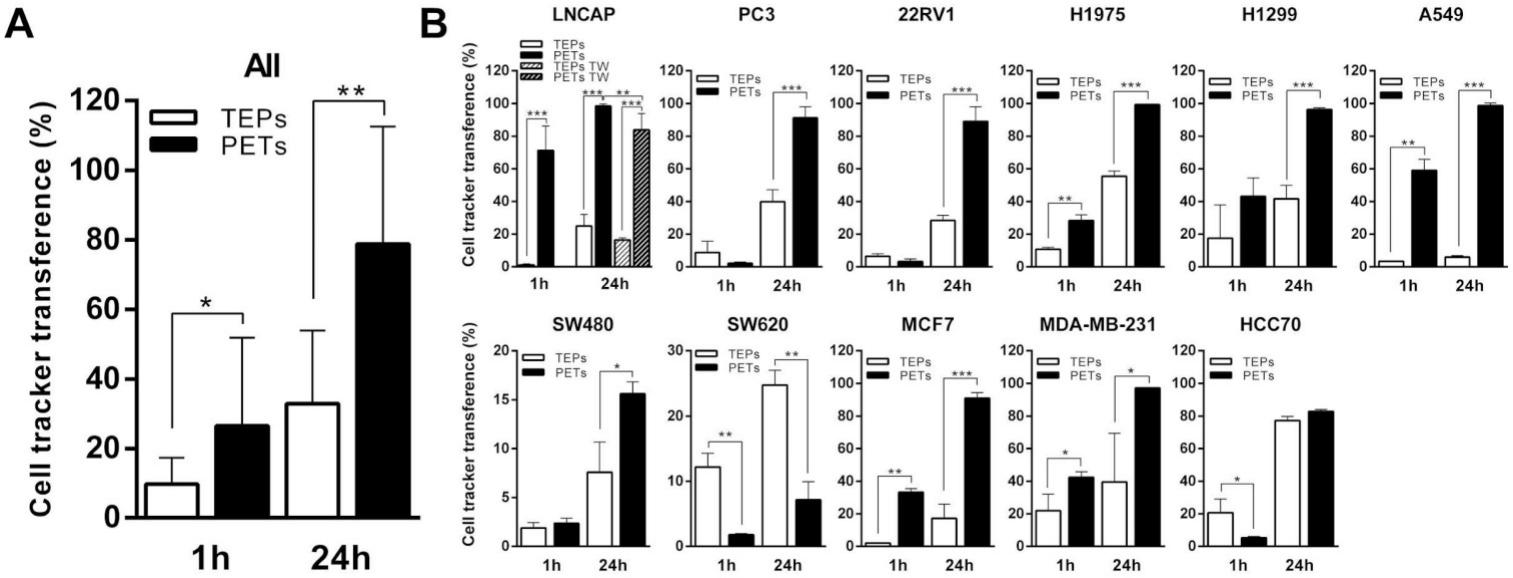
** Bidirectional lipids interchange between platelets and tumor cells in co-culture.** (**A**) Pooled and (**B**) individual results (B) for each cell line transference of lipid cell tracker from cells to platelets in white (TEPs) and from platelets to cells in black (PETs) after 1h and 24h of co-culture. Student's *t*-test, was performed, only significant results are presented (*p<0.05; **p<0.01; ***p<0.001). TW: Transwell membrane. TEPs: Tumor-educated platelets; PETs: Platelets-educated tumor cells.

Cancer cells were pre-labeled using a lipophilic DiO cell tracker and the percentage of CD42^+^ platelets acquiring fluorescence after 1 h and 24 h of co-culture is presented as tumor-educated platelets (TEPs). Alternatively, platelets were pre-labeled with DiO cell tracker and the percentage of EpCAM^+^ tumor cells that acquired fluorescence is shown as platelets-educated tumor cells (PETs). Although the transference of material from tumor cells to platelets has been extensively studied, our grouped analysis of the interaction between all different tumor cell lines and platelets (Figure 1A) showed that only 9.69 ± 2.3% and 32.95 ± 6.33% of the platelets had receive lipid cell tracker from cells at 1 h and 24 h, respectively; while 26.54 ± 7.64% and 78.77 ± 10.17% of tumor cells showed lipid cell tracer from platelet transference at 1 h and 24 h, respectively. Individual results for each cell line are presented in Figure 1B. All cell lines, excluding SW620 and HCC70, showed higher lipid transference from platelets to cells than from cells to platelets at 24 h of co-culture. Lipid transference through transwell membrane was studied in LNCAP cells, and no significant differences were found (*p*=0.079) for cell tracker transference between cells to platelets, suggesting cell contact is not mandatory; however, our results showed a significant reduction of platelet-to-cell lipid transference (83.8 ± 5.02%) compared with the co-culture control (98.38 ± 0.56%) (*p* = 0.007) (Figure 1B). These results might suggest that transference from platelets to cancer cells is more dependent on cell contact than the one from cells to platelets.

### Transference of lipid components from platelets to cells is mediated by different mechanisms

**Figure 2:**
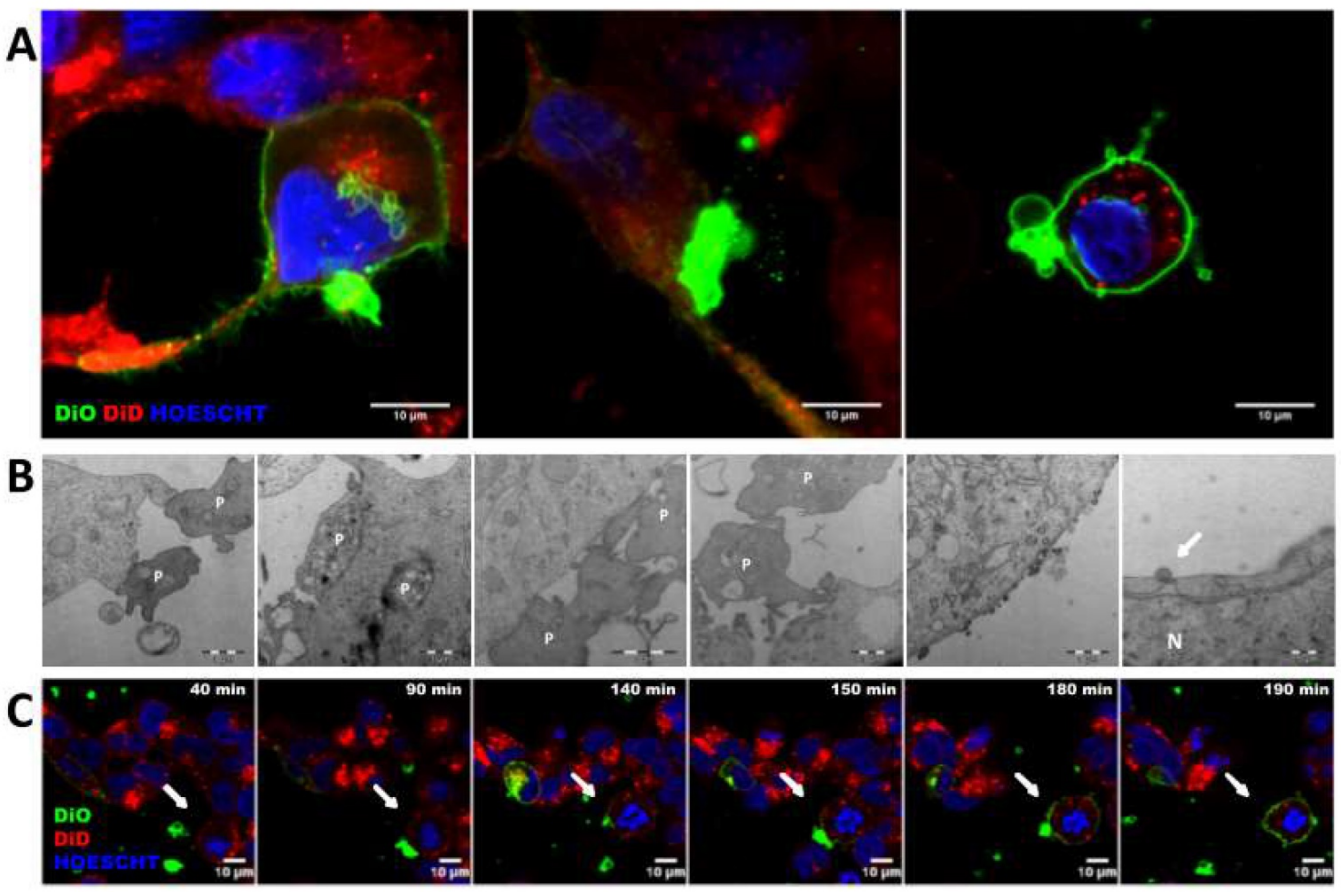
** Platelets and tumor-cells interactions.** (**A**) Membrane cell tracker transference by direct contact (left), platelet-microparticles release (center) and platelet cell tracker transference to cell nucleus envelope (right). (**B**) Electron microscopy pictures from left to right: platelet-cell membrane fusion, platelet internalization, platelet-cell contact and platelet microparticles delivery, directional microparticles platelet release, microparticle fusion with cell membrane and microparticle fusion with plasmatic membrane and nucleus (N) envelope. P indicates platelets. (**C**) Representative pictures of time-lapse imaging of platelets (green) transferring cell tracker to tumor cell (red) membrane. Arrows point cells of interest.

The mechanisms involved in transference from platelets to tumor cells were exhaustively analyzed through confocal and electron microscopy. As visualized, platelets were able to modify tumor cell membrane through direct contact, observing that the tumor cell membrane became green after acquisition of the lipid DiO green marker. In particular, platelets were able to fuse with the tumor cell membrane (Figure 2A and Figure C, Figure S4), to be integrally internalized (Figure 2A and Figure 2B) and then, to fuse with the cell nuclear envelope (Figure 2A, middle and Figure 2B right). Additionally, platelets were able to transfer information through vesicles (Figure 2A right and Figure 2B center).

### Cancer cells and platelets transfer RNA components

**Figure 3:**
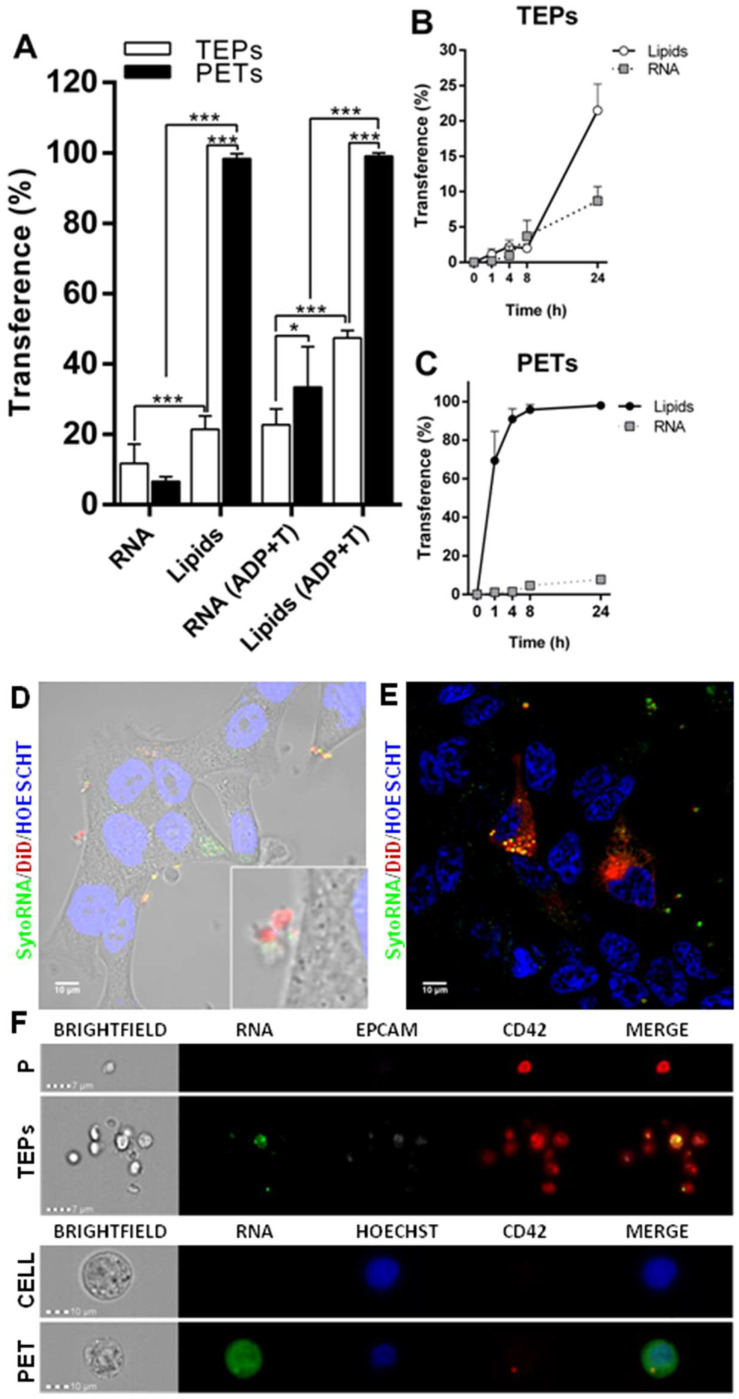
** Structural and RNA transference between platelets and LNCAP prostate cancer cell line in co-culture.** (**A**) Transference of lipids and RNA labeled molecules from cells to platelets (TEPs). Two-way ANOVA (Tukey's multiple comparison test) (*p<0.05, **p<0.01, ***p<0.001). (**B**) Kinetics of cells trackers transference from cell to platelets and (**C**) from platelets to cells. (**D-E**) Detail of RNA transference from platelets to cells. (**F**) ImageStream example pictures of platelets (P) and TEPs and tumor cell (Cell) and PETs after RNA tracker transference. TEPs: Tumor-educated platelets; PETs: Platelets-educated tumor cells.

Transference of other cellular components as RNA was also analyzed, by using a RNA labeling method (SytoRNA) in LNCAP cells. After 24 h of co-culture, tumor cells had transferred SytoRNA-labeled RNA to 11.47 ± 2.08% of the platelets while lipid cell tracker was transferred to 21.47 ± 1.53%. In the opposite direction, platelets transferred SytoRNA-labeled RNA to 6.62 ± 0.59% of tumor cells whereas 98.38 ± 0.56% showed lipid uptake at 24 h. RNA transference efficiency was significantly lower than lipid transference, in both TEPs (*p* < 0.001) and PETs (*p* < 0.001) (Figure 3A) and there was no difference in RNA transference between cell to platelets and platelets to cells (Figure 3A-C). Interestingly, pre-treatment of platelets with ADP+Trombin significatively increased transference from cells to platelets of DiO cell tracker and SytoRNA-labeled RNA (*p* < 0.01 and *p* < 0.001, respectively). Pre-activation of platelets also increased RNA transference from platelets to cells (*p* < 0.001) while no differences were found in DiO cell tracker transference from platelets to cells independently on whether they were activated or not (Figure S3B). Thus, it was observed that after ADP+T pre-treatment of platelets, RNA transference was more efficient from platelets to cells than from cells to platelets (p < 0.05) (Figure 3).

SytoRNA-labeled RNA delivery was also analyzed using imaging techniques, showing RNA release in platelet-derived microparticles and platelet-labeled RNA inside tumor cells (Figure 3D and Figure 3E). ImageStream pictures showed both SytoRNA-labeled RNA transference from tumor cells to platelets (TEPs) and from platelets to tumor (PETs) (Figure 3F and Figure S5).

### Tumor cells and platelets exchange proteins

**Figure 4:**
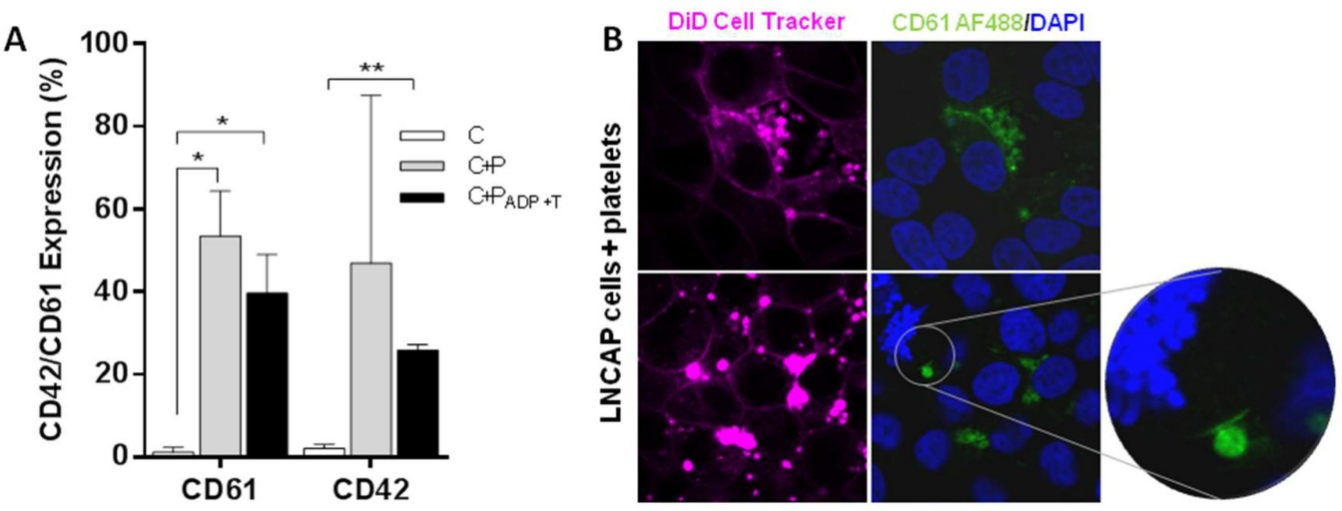
** Transference of platelet proteins to tumor cells. (A)** CD61 and CD42 transference from platelets to tumor cells after 24 hours of co-culture. Cells cultured alone (C), cells and platelets (C+P) and cells cultured with ADP+T treated platelets. Student's *t*-test was performed comparing all groups. Only significant results are shown (*p<0.05, **p<0.01). **(B)** Representative examples of CD61 transference from platelets to LNCAP cell in culture.

Protein transference analysis was observed on ImageStream experiments revealing that not only tumor cells are able to transfer a protein of epithelial origin (EpCAM) to platelets but also platelets transfer a private protein (CD42) to tumor cells (Figure 3F and Figure S5B). After that, platelets specific proteins (CD42 and CD61) were analyzed by flow cytometry in a prostate cancer cell line alone (C), after co-culture with platelets (C+P), and after co-culture with activated platelets [C+P_(ADP+T)_]. We observed CD61 and CD42 expression in more than 40% of EpCAM^+^ tumor cells after 24 h of co-culture with platelets while tumor cell culture alone did not show any expression of these markers. Interestingly, pre-activation of platelets did not enhance protein transference from platelets to tumor cells (Figure 4A). Imaging of platelets to tumor cell transference confirmed cellular uptake of CD61 and incorporation to their membranes (Figure 4B).

### Platelets-specific proteins are detected in a subpopulation of circulating tumor cells from prostate cancer patients

**Figure 5:**
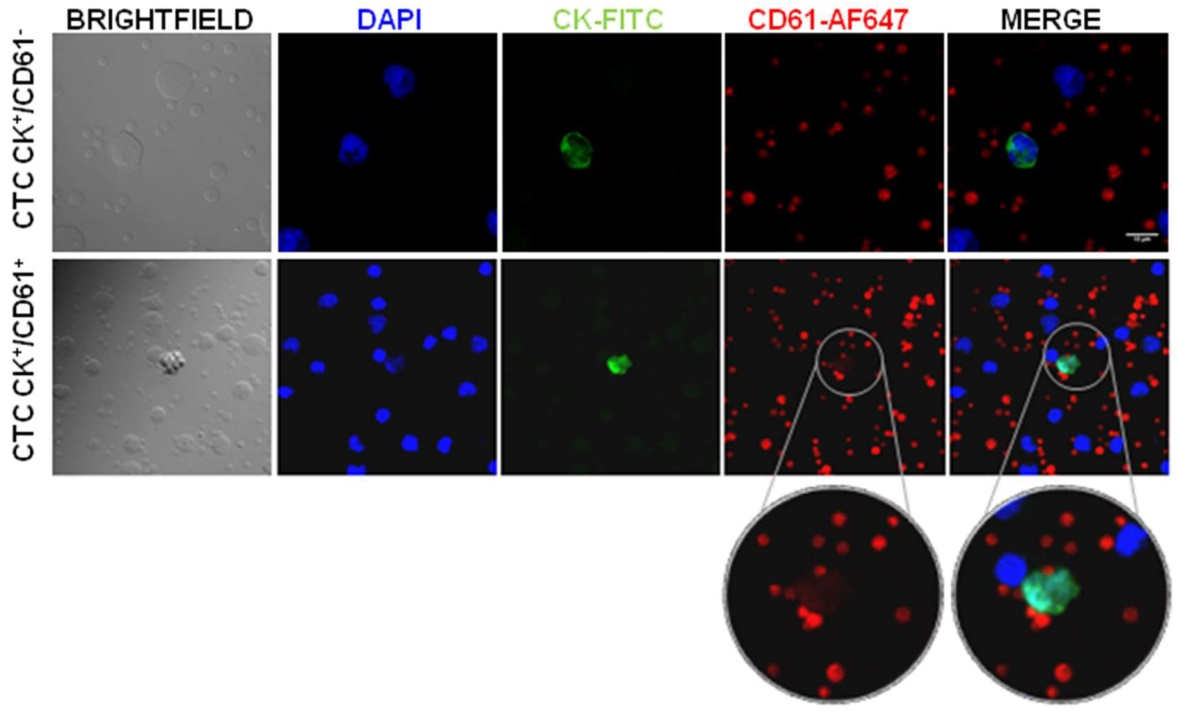
** Heterogeneity of CD61 expression in Circulating Tumor Cells (CTCs) from prostate cancer patients.** Confocal microscopy images of CTCs positive for CK (FITC.green) and nucleus (DAPI. blue). CD61 platelet marker absent and present in CTCs (AF-647. red). Abbreviations: CK: cytokeratin, AF: Alexa Fluor.

In order to confirm CD61 transference, we isolated circulating tumor cells (CTCs) from peripheral blood samples from 4 advanced and 11 localized prostate cancer patients and analyzed the expression of CD61. Our results showed presence of two CTCs subpopulations according to CD61 expression (CK^+^/CD61^+^ and CK^+^/CD61^-^), as well as intra and inter patient heterogeneity (Figure 5). Interestingly, all CTCs analyzed in the advanced prostate cancer stage presented CD61^+^ expression while three localized stage patients showed CTCs with absence of CD61 expression (CK^+^/CD61^-^) (Table 1).

### Tumor cells and platelets cross-talk induce tumor cell plasticity

**Figure 6:**
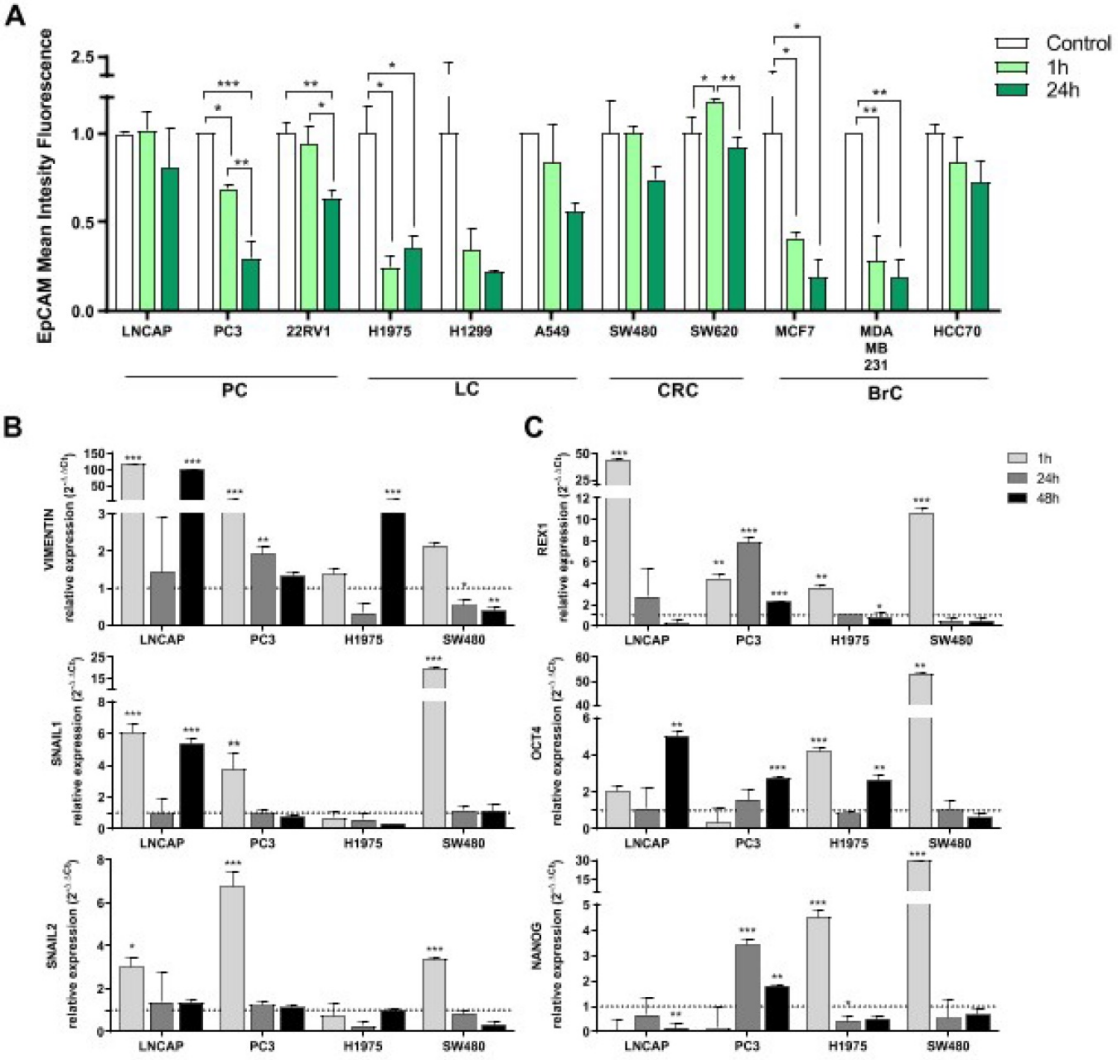
** Influence co-culture with platelets EMT and pluripotency.** (**A**) EpCAM expression levels after co-culture with platelets are shown as relative mean intensity fluorescence by flow cytometry. Data were normalized to cell cultures alone for each cell line (Control). One-way ANOVA was performed, only significant results are presented (*p<0.05; **p<0.01; ***p<0.001. Abbreviations are: (PC) prostate cancer, (BrC) breast cancer, (LC) lung cancer, (CRC) colorectal cancer. (**B**) Gene expression of EMT related genes and (**C**) pluripotency related genes. RT-qPCR data is presented as relative expression to culture cells alone. Dotted lines in Y axis represent normalized value for cells cultured alone. Student's *t*-test was performed comparing co-culture and cells cultured alone at each time point. Only significant results are shown (*p<0.05, **p<0.01, ***p<0.001).

Consequences in tumor cell behavior after interaction with platelets was studied in terms of EpCAM protein expression, gene expression of EMT and stemness markers and cell growth. Expression levels of EpCAM were analyzed in tumor cell lines 1 h and 24 h after co-culture with platelets. There was a clear trend of EpCAM expression reduction after 1 h of co-culture with platelets in all tumor types analyzed (prostate, lung, colorectal and breast), being significant in PC3, 22RV1, H1975, MCF7 and MDA-MB-231 cell lines. This reduction was greater after 24 h of co-culture (Figure 6A).

Induction of the EMT process and acquisition of stem-like features by tumor cells after platelet interactions were further analyzed. We observed a significant induction of the EMT process (upregulation of *VIMENTIN*, *SNAIL1*, *SNAIL2)* at short time-point (1 h) in LNCAP, PC3 and SW480 cells lines and also at 48 h in LNCAP cells (for *VIMENTIN* and *SNAIL1*). In contrast, H1975 cell line from lung cancer only induced *VIMENTIN* expression at 48 h of co-culture (Figure 6A). Regarding stemness induction, expression of *REX1* gene was increased after 1 h of platelet co-culture in all cell lines, whereas *OCT4* and *NANOG* were only increased in H1975 and SW480 cell lines at short term. *OCT4* was induced at 48 h of co-culture in LNCAP, PC3 and H1975 cell lines (Figure 6B).

Moreover, we studied the effect of platelets addition to prostate cancer cell culture in tumor cell growth. Seven of the eleven (63.6%) cell lines studied showed a significant increase in cell proliferation after 48 h of co-culture with platelets suggesting that the interaction with platelets induced a more proliferative tumor cell phenotype in several tumor types (Figure S6).

Interestingly, we observed that while addition of platelets did not involve special protection against apoptosis or cell death for the 22RV1 cell line at 24 h in standard culture conditions (10% FBS), a significant reduction of cell death and apoptosis was found for this tumor cell line when co-culturing with platelets in stress/starving conditions (1% FBS) during 24 h (Figure S7).

Wound healing assays did not show significant differences between prostate cancer cell lines cultured alone and co-cultured with platelets (Figure S8).

## Discussion

Over the last several years accumulating evidence demonstrated that platelets exert several additional biological functions beyond limiting blood loss and promoting wound healing recognizing their role on tumorigenesis 12. To describe the interaction between tumor cells and platelets, the term tumor-educated platelets (TEPs) was coined identifying a novel biomarker that enables blood-based cancer diagnostics and treatment monitoring 23. However, complex bidirectional interactions essential for cancer progression, occur between tumor cells and platelets and involve direct contact through the formation of tumor-platelet aggregates and release of soluble factors 8,24. Our study shows that platelets transfer lipids, proteins and RNA to tumor cells inducing structural, genetic and functional modifications to the tumor cells.

A large body of literature related with the role of platelets in cancer has been particularly focused on their ability to facilitate circulating tumor cells (CTCs) extravasation and to protect them from shear forces and assault of natural killer (NK) 25,26. However, most of these studies do not describe structural (lipids and protein) transference promoting tumor cell membrane modifications and not only platelet cloaking 27. Here, for the first time, we demonstrated that platelets are able not only to modify lipid composition of cell membrane but also to introduce themselves inside tumor cells and to modify the lipids of the nuclear envelope.

Some recently published works 28-30 showed that microparticles (or microvesicles) derived from platelets infiltrate the tumor, being able to transfer miRNAs to tumor cells. In this context, we observed that platelets were able to transfer RNA to tumor cells by direct contact and by the release of microparticles containing RNA. Our results coincide with results obtained by Risitano et al. who observed the ability of platelets to transfer RNA to leukocytes in mouse models and to vascular cells in culture 31.

Regarding protein transference, according to previous results, we detected that cancer cells became positive to beta-3 integrin markers (CD61 or gpIIIa) after co-culture 15. Importantly, we found a subpopulation of CK^+^/CD61^+^ CTCs isolated from prostate cancer patients suggesting these CTCs might have been in touch with platelets in circulation. In a recent paper by Jiang X, et al. the importance of including platelet markers for improving CTCs isolation was analyzed 32. According to them, the platelet-targeted isolation methodology would be applicable to CTCs of both epithelial and mesenchymal phenotypes. Even though this work analyzed platelets markers, unlike ours, their interest was to detect CTCs covered by platelets to identify a subpopulation of CTCs difficult to isolate with the current technologies. This finding is especially important since CD61 has a main role in reprogramming and re-educating the tumor microenvironment and is essential for the EMT process, stemness regulation, and drug resistance acquisition 33. Therefore, presence of this integrin in CTCs might identify subpopulations with increased migration properties, progression potential, and resistance to different treatments, what in turn may pinpoint metastatic CTCs disguised as platelets.

The EMT is another key process analyzed in this study as changes in the lipid composition of the cell membrane might induce this mechanism. The EMT involves loss of EpCAM expression together with an increase of mesenchymal-associated gene expression 34,35. In accordance to that, we found an increase of *VIMENTIN* and *SNAIL* gene family expression in tumor cell lines after co-culture with platelets. Importantly, these modifications varied regarding tumor type, most likely due to constitutive expression of EMT markers in some tumor types (such as lung cancer cell lines, that present a semi-mesenchymal phenotype) 36. In addition, EMT is known to be related with stem-like phenotype, which is also associated with drug resistance and disease progression 37. In our work, we found a similar induction pattern of progenitor gene expression (*REX1, NANOG* and *OCT4*) and EMT gene expression patterns induced by platelets co-culture.

Therefore, alterations in cell and nuclear membranes may affect processes as relevant as cell cycle and genome regulation, cell signaling, or migration and metastasis 38,39.

Interestingly, our results showed alterations of cancer cell membranes after platelet-cell interaction, promoting changes in cell functionality. We observed that co-culture with platelets induced alterations in cell growth compared with naïve tumor cells.

Likewise, several studies described platelet interactions with many blood cell types, including CTCs, leukocytes, and endothelial cells 6,40. However, the mechanisms by which platelets promote CTC survival in the bloodstream are not fully understood yet. Thus, some studies analyzed the role of platelets on metastasis demonstrating that platelets activation promotes tumor cells survival through thrombin expression, increasing their metastatic potential 25. Packle T et al. demonstrated that platelet coating may cause transference of MHC class I to tumor cell surface resulting in high expression levels of platelet-derived normal MHC class I, which in turn, mimics host cells and helps them escaping immune surveillance 15. In that sense, we observed that under stressful conditions, platelets protect tumor cells from apoptosis and cell death. This result is interesting, since CTCs are exposed to different stress factors (including oxidative stress, anoikis) 41. Therefore, it is reasonable to think that the direct interaction and transference of biomolecules from platelets to CTCs has an important role in the survival of these CTCs in the bloodstream.

Thus, despite our data being in accordance with previous published results, our work extensively describes the mechanisms by which bi-directional transference occurs and quantifies transference of three different groups of molecules (lipids, proteins and RNA) between platelets and tumor cells from several tumor types.

## Conclusions

In conclusion, to the best of our knowledge this is the first work in which tumor cells and platelets cross-talk (focusing on tumor cells modifications induced by platelets) is described and analyzed in such a comprehensive manner. We demonstrated that the role of platelets goes far beyond indirect activation of pathways associated with the microenvironment to support CTC dissemination from the primary tumor. Our data suggest that platelets confer cell plasticity, modifying tumor cell behavior, promoting cell growth and CTC survival, allowing them to evade the immune system and probably chemotherapy. This biological exchange promotes presence of CTC subpopulations disguised as platelets with potentially more aggressive phenotypes. From our point of view, a deeper analysis of these interactions, including *in vivo* experiments and CTCs characterization in large cohorts is needed to reach a better knowledge of their biological and clinical consequences.

## Supplementary Material

Supplementary methods, figures 1-3 and 5-8, and tables.

Supplementary figure 4 (time lapse video).

## Figures and Tables

**Table 1:** Circulating tumor cell enumeration and characterization according to prostate cancer stage

Stage	Patients	CTC/10ml	
		CK^+^/CD61^+^	CK^+^/CD61^-^
Advanced	1	5	0
	2	3	0
	3	2	0
	4	0	0
Localized	5	3	0
	6	2	1
	7	2	1
	8	1	1
	9	1	0
	10	0	0
	11	0	0
	12	0	0
	13	0	0
	14	0	0
	15	0	0

## Abbreviations

CK: Cytokeratin
CTCs: Circulating tumor cells
EMT: Epithelial-to-mesenchymal transition
PETs: Platelet-educated tumors
TEPs: Tumor-educated platelets
