# Supplementary material for: Exchange of cellular components between platelets and tumor cells: impact on tumor cells behavior
Source: Theranostics. 2022 Feb 7;12(5):2150–61. doi: 10.7150/thno.64252 (PMC8899588; doi:10.7150/thno.64252)

Supplementary Information

Exchange of cellular components between platelets and tumor cells: impact on tumor cells behavior

Alba Rodriguez-Martinez[1,2]†, Iris Simon-Saez[1,3]†, Sonia Perales[1,3], Carmen Garrido-Navas[1], Alessandro Russo[4], Diego de Miguel-Perez[4], Ignacio Puche-Sanz[1,5], Clara Alaminos[6], Jorge Ceron[1,3], Jose A. Lorente[1,2], Maria Pilar Molina[1], Coral Gonzalez[1], Massimo Cristofanilli[7], Alba Ortigosa-Palomo[1], Pedro J. Real[1,3,8], Christian Rolfo[4*], María J. Serrano*[1,9,10]

Supplementary Methods

Cell Proliferation

The effect of co-culturing platelets with tumor cells on cell proliferation was evaluated with the real-time cell monitoring assay (RTCA) (xCELLigence; ACEA Biosciences, Inc.). Cells were seeded in the RTCA plates and isolated platelets were added 24 h afterwards. Cell growth was monitored from seeding in a 4 h interval on the RTCA system for 72 h and impedance was recorded as a measurement of Cell Index (CI). Experiments were performed in quadruplicates and outliers were removed.

Cell death and apoptosis

Platelets were obtained by centrifugation from peripheral blood samples from the same healthy donor. In standard condition, prostate cancer cells were seeded as described previously in 12-well plates, platelets were added at physiological concentration (150,000 plt/µL), time of addition is considered time 0. The effect on cell death and apoptosis at different times (24 h, 48 h and 72 h) was evaluated by flow cytometry analyzing the incorporation of 7AAD and expression of Annexin V, according to manufacturer recommendations. For stress conditions (1% FBS), 24 h after cells seeding, culture media (10 % FBS) were replaced by 1 % FBS, cells were cultured 24 h in this condition, after that platelets were added in 1 % FBS, and experiment were carried out in this conditions.

Wound healing assay

Prostate cancer cell lines were cultured as previously described for 1 % FBS conditions. Wound was performed using 100 µL tip, wells were washed twice with PBS, platelets were added at physiological concentration (150,000 plt/µL), time of addition is considered time 0. Wound area was calculated using ImageJ, experiments were performed in triplicate.

Supplementary Tables

Table S1. Tumor cell and platelet molecules selected for cell characterization and transference studies.

	Putative Location	Description	Rationale selection	Application
**Epithelial cell adhesion molecule (EpCAM) / CD326**	Cell surface of epithelial cells	Transmembrane glycoprotein involved in adhesion, migration, signalling, etc	Widely studied protein in epithelial tumor characterization. Easily detectable on the surface	Tumor cell to platelets transference experiments (Flow cytometry, Image Stream)
**Pan-keratin (Cytokeratins 7, 8, 17 and 18)**	Intracytoplasmic cytoskeleton of epithelial cells	Component of intermediate filaments of cytoskeleton	Stable protein during EMT process of tumor cells, used routinely in CTC isolation and detection	CTC isolation and detection (Confocal microscopy)
**Integrin IIb / CD41a**	Cell surface of platelets and megakaryocytes, mainly	Form CD41/CD61 complex	Specific platelet antigen	Platelet activation experiments (Flow cytometry)
**Glycoprotein Ib / GPIb / CD42b**	Cell surface of platelets and megakaryocytes, mainly	CD42b and CD42c form Von Willebrand factor receptor	Specific platelet antigen	Platelet to tumor cell transference experiments (Flow cytometry, Image Stream)
**Integrin beta-3 (β3)/CD61**	Cell surface of platelets, megakaryocytes, macrophages, endothelial cells among others.	Form CD41/CD61 complex in platelets. CD51/CD61 complex in other cell types	Specific platelet antigen	CTC characterization (Confocal microscopy) Platelet to tumor cell transference experiments (Flow cytometry)
**PAC-1**	Cell surface of platelets	Activation-induced conformational epitope PAC-1 on CD41/CD61 complex	Specific marker of platelet activation	Platelet activation experiments (Flow cytometry)

Table S2. Primer sequences used for RT-qPCR in this study.

	Forward Sequence (5´-3´)	Reverse Sequence (5´-3´)	Product length (pb)
**VIMENTIN**	CCAGGCAAAGCAGGAGTC	CGAAGGTGACGAGCCATT	213
**SNAIL1**	GCTGCAGGACTCTAATCCAGA	ATCTCCGGAGGTGGGATG	84
**SNAIL2**	TGGTTGCTTCAAGGACACAT	GCAAATGCTCTGTTGCAGTG	77
**REX1**	CAGATCCTAAACAGCTCGCAGAAT	GCGTACGCAAATTAAAGTCCAGA	306
**OCT4**	AGTGAGAGGCAACCTGGAGA	ACACTCGGACCACATCCTTC	110
**NANOG**	TGCAGTTCCAGCCAAATTCTC	CCTAGTGGTCTGCTGTATTACATTAAGG	92
**GAPDH**	TGCACCACCAACTGCTTAGC	GGCATGGACTGTGGTCATGAG	87

Supplementary Figures

Figure S1. Methods summary.

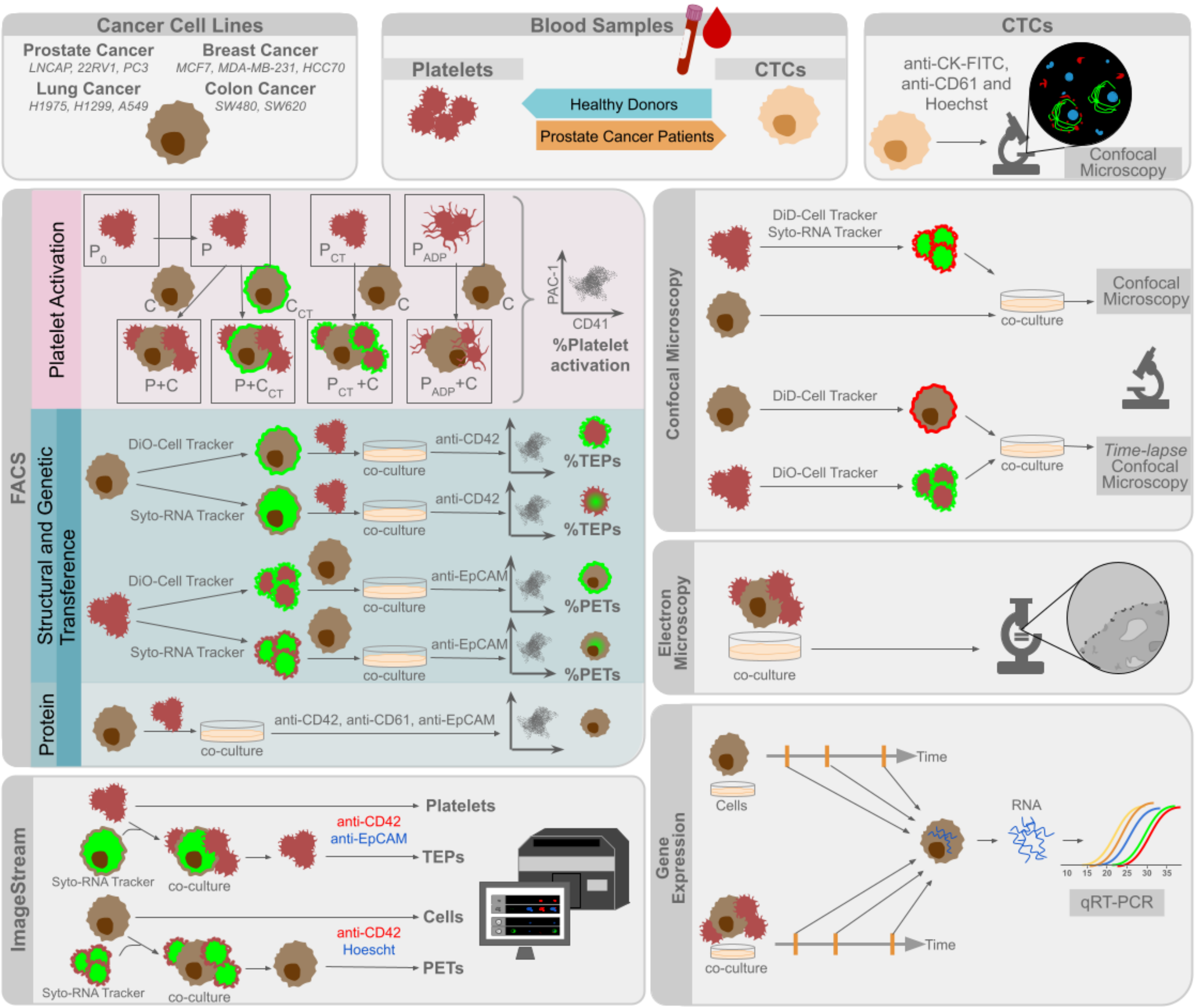

Figure S2. Platelets and LNCAP prostate cancer cell line co-culture analysis gates. (**A**) Gates used for analysis of the platelets activation or (**B**) the transference of structural cell tracker from cells to platelets and (**C**) platelets to cells. All the gates were taken from a negative control.

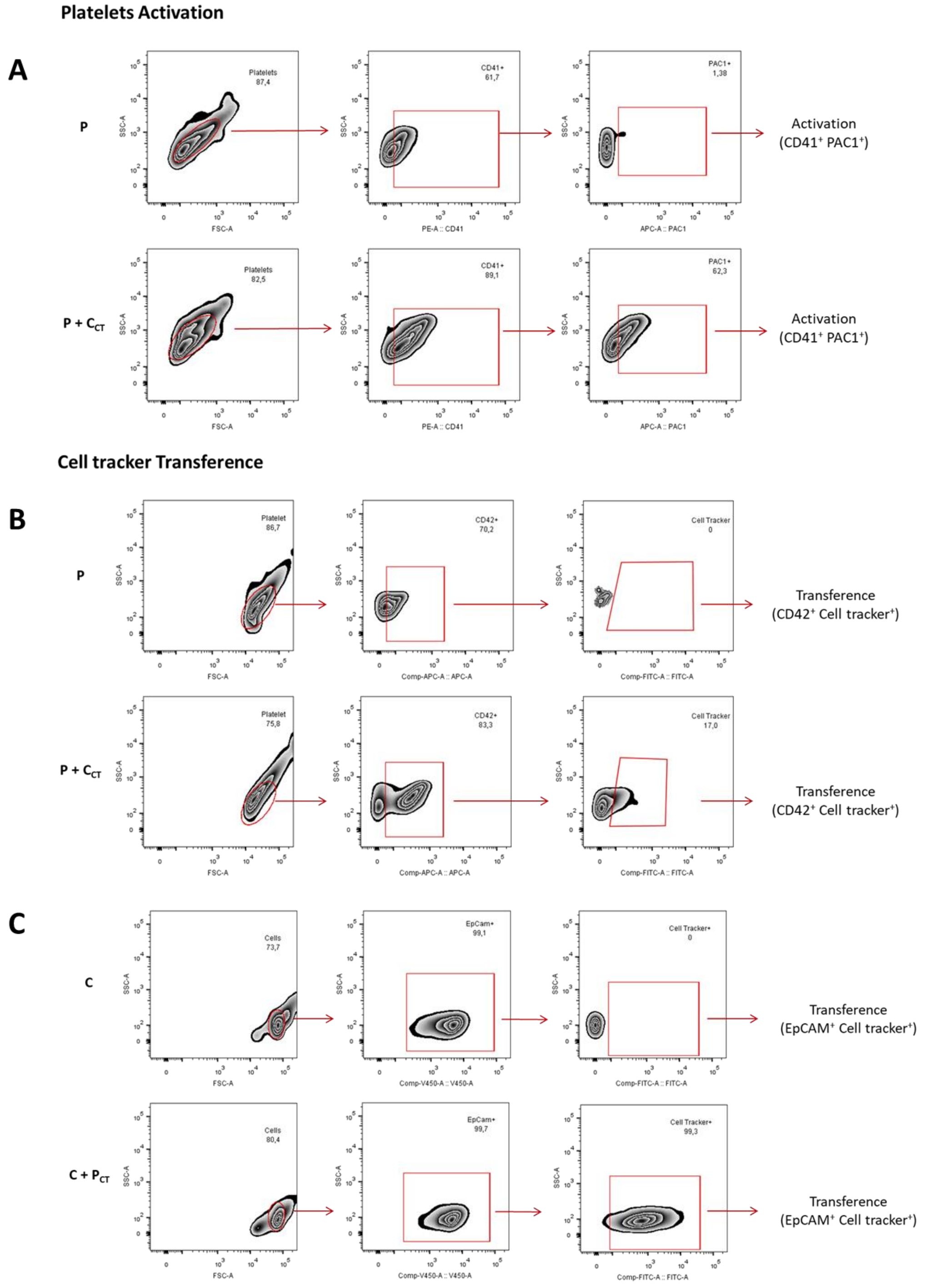

Figure S3. Co-culture effects on platelet activation. (**A**) Platelet activation was presented as percentage (%) of PAC-1^{+} platelets. White bars showed platelets cultured alone: after isolation: (P_0) non-activated platelets; after 24 h in culture: (P) untreated platelets, (P_{CT}) cell tracker-labeled platelets, (P_{ADP+T}) ADP+Thrombin-treated platelets. Black bars showed platelets after 24 h co-culture with LNCAP cells: (P + C) platelets with unlabeled cells, (P + C_{CT}) platelets with cell tracker-labeled cells, (P_{CT} + C) cell tracker-labeled platelets with unlabeled cells, and (P_{ADP+T} + C) ADP+Thrombin-treated platelets with unlabeled cells. Data is presented as means ± SD. One-way ANOVA (Dunnett's multiple comparisons test) were performed for all conditions *vs* control (P_0), (ns: no significant; * $p < 0.05$; **$p < 0.01$; ***$p < 0.001$). **B**) Transference of Lipids and RNA between resting or ADP+Trombin (ADP+T) platelets and LNCAP cells. Two-way ANOVA (Tukey's multiple comparison test) A (*p < 0.05, **p < 0.01, ***p < 0.001). TEPs: Tumor-educated platelets; PETs: Platelets-educated tumor cells.

A **B**

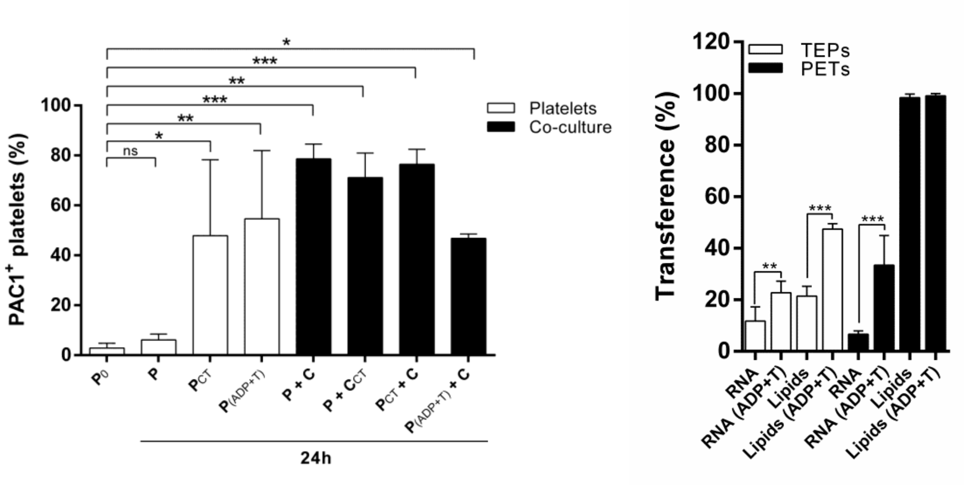

Figure S4. Confocal Time Lapse of platelet and LNCAP co-culture. Platelets were labelled with DiO-cell tracker (green), cells with DiD-cell tracker (red) and DNA was stained with Hoechst (blue).

Figure S5. ImageStream gallery. (A) Tumor educated platelets (TEPs) identified with CD42 (red) showing EpCAM (blue) and Syto RNASelect-RNA (green) uptake from tumor cells. **(B)** Platelet-educated tumor cells (PETs) labelled with Hoechst (blue) showing Syto RNASelect labelled RNA (green) and CD42 (red) acquired from tumor cells.

A

B

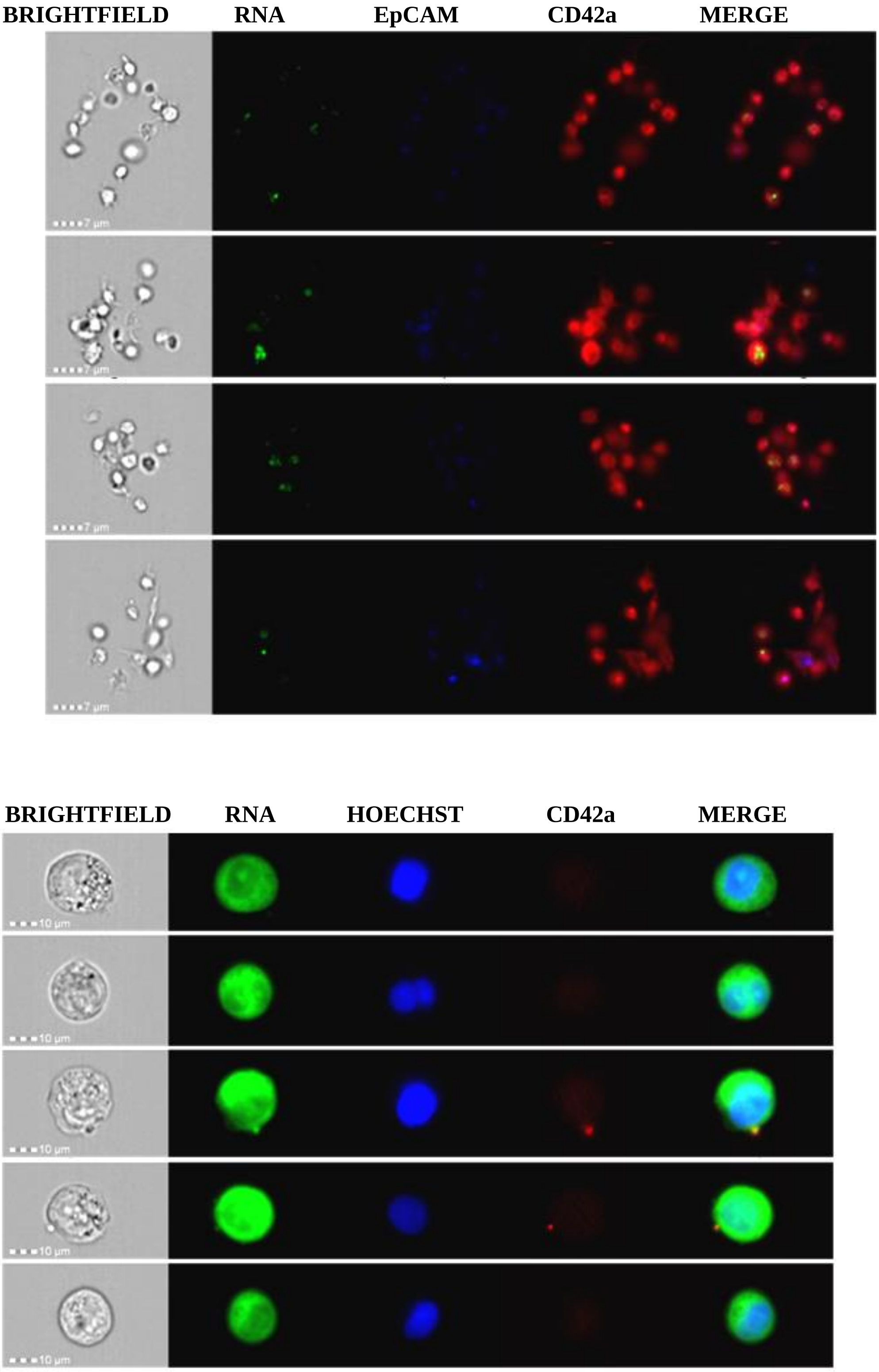

Figure S6. Cell growth of cancer cell lines alone (black line) or in co-culture with platelets (grey line). Cell growth was measured during 72 h, platelet addition was considered time 0. Impedance of cell cultured alone (C) at the endpoint was considered 100% for normalization. Two-way (Sidak's multiple comparisons test) ANOVA for 24 h and 48 h of co-culture is shown (ns: no significant; *$p < 0.05$; **$p < 0.01$; ***$p < 0.001$).

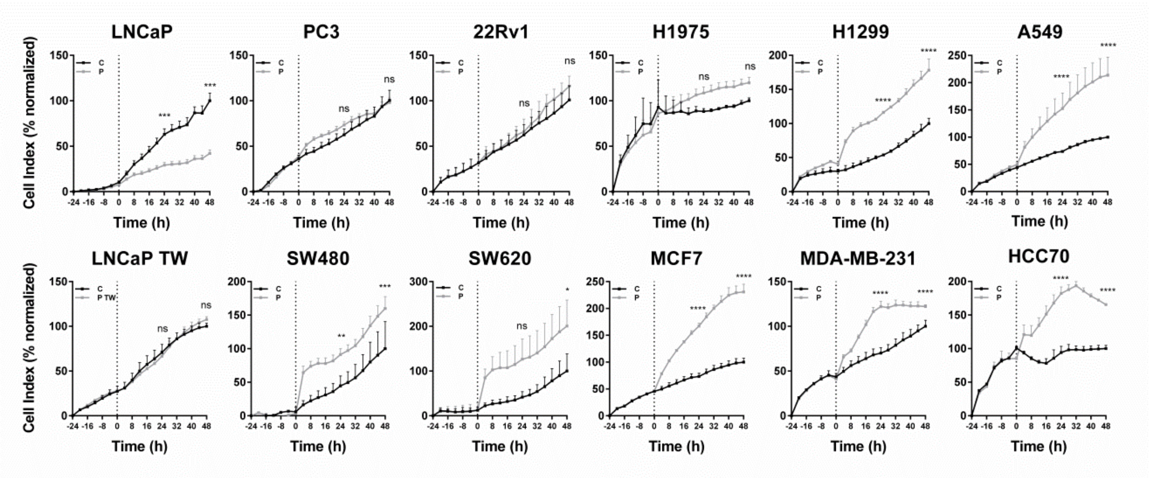

Figure S7. Cell death (7AAD^{+}) and apoptosis (Anexin V^{+}) of prostate cancer cell lines cultured alone (white bars) and co-cultured with platelets (black bars) in 10 % of FBS (A) and 1 % of FBS. T-student test (*$p < 0.05$, only significant results are shown).

A)

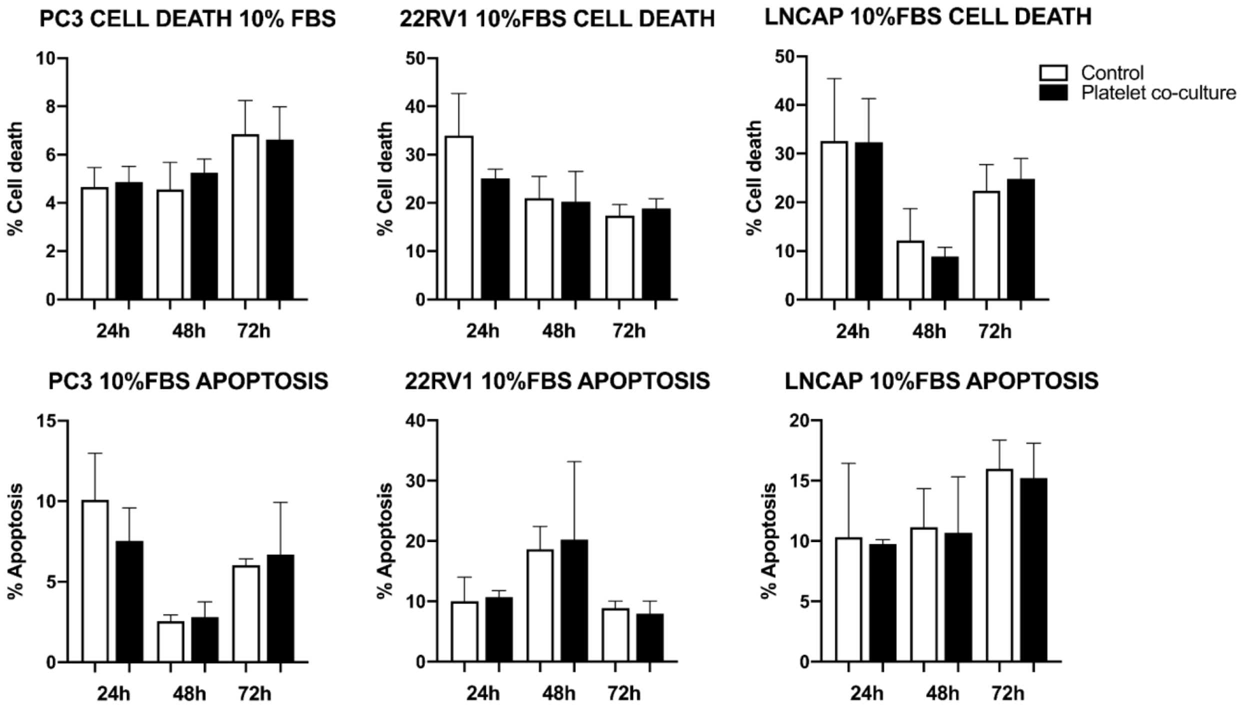

B)

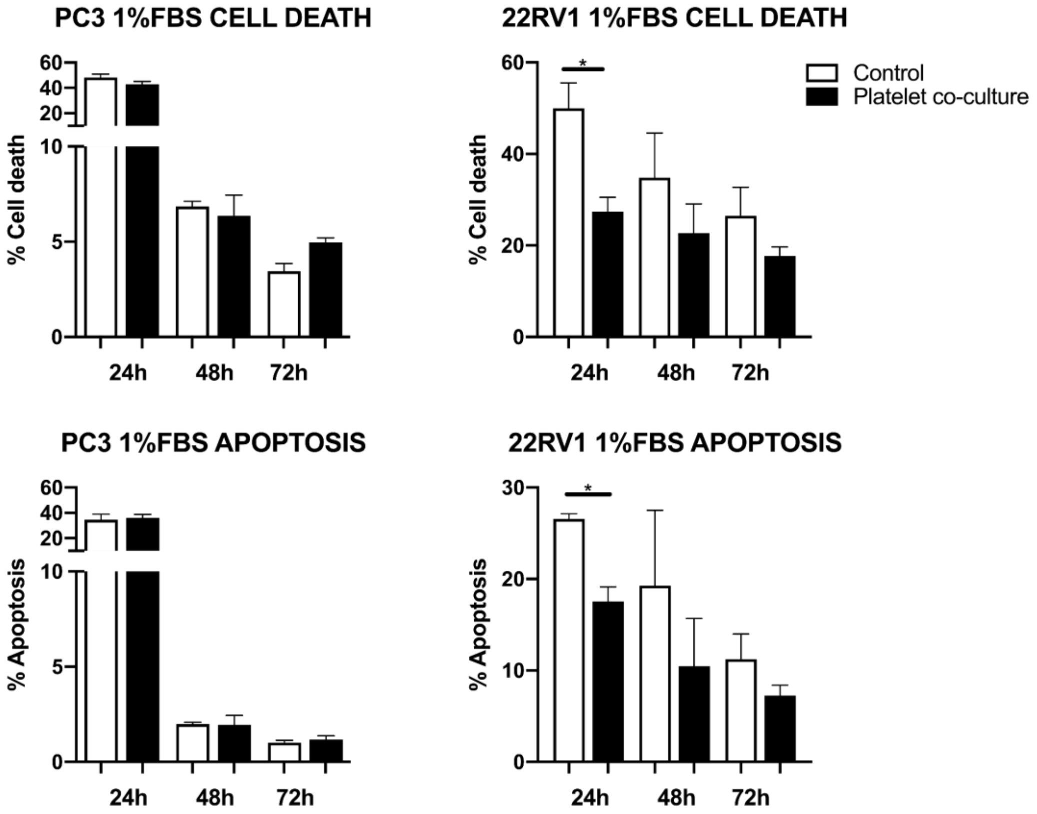

Figure S8. Wound healing assay. A-B) Normalized percentage of wound area at different time points. T-student test (ns: no significant). C-D) Representative pictures of wound healing assay, measured area (green lines).

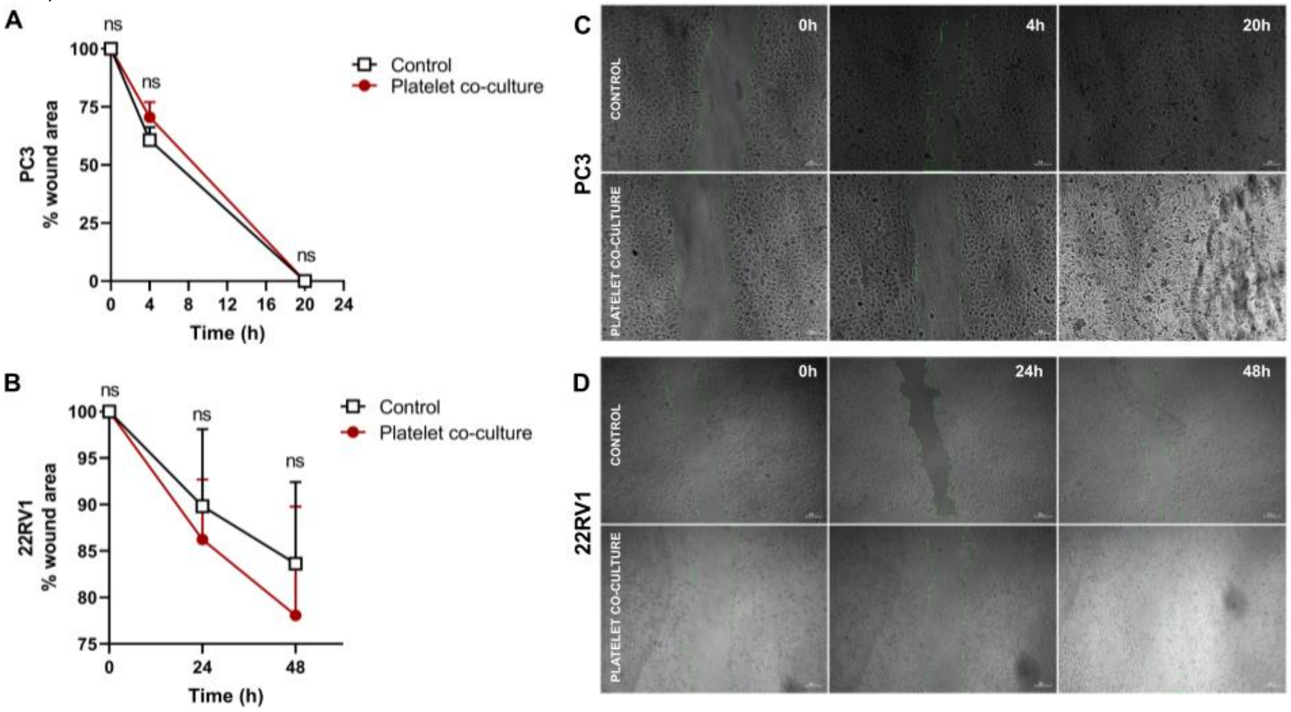

Supplement: Supplementary file 1 — Supplementary methods, figures 1-3 and 5-8, and tables. [file thnov12p2150s1.pdf]
